# Application of a Scorecard Tool for Assessing and Engaging Media on Responsible Reporting of Suicide-Related News in India

**DOI:** 10.3390/ijerph18126206

**Published:** 2021-06-08

**Authors:** Lakshmi Vijayakumar, Manisha Shastri, Tanya Nicole Fernandes, Yash Bagra, Aaryaman Pathare, Arpita Patel, Padam Jain, Yesha Merchant, Gregory Armstrong, Soumitra Pathare

**Affiliations:** 1 Department of Psychiatry, Voluntary Health Services and SNEHA Suicide Prevention Centre, Tamil Nadu 600017, India; 2 Centre for Mental Health Law and Policy, Indian Law Society, Pune 411004, India; manisha@cmhlp.org (M.S.); tanya@cmhlp.org (T.N.F.); yashbagra@gmail.com (Y.B.); pathareaaryaman@gmail.com (A.P.); arpita@cmhlp.org (A.P.); jpadam123@gmail.com (P.J.); merchantyesha23@gmail.com (Y.M.); spathare@cmhlp.org (S.P.); 3 Melbourne School of Population and Global Health, Nossal Institute for Global Health, University of Melbourne, Melbourne 3010, Australia; g.armstrong@unimelb.edu.au

**Keywords:** suicide, suicide prevention, media reporting of suicide, WHO guidelines on responsible reporting of suicide, media and suicide

## Abstract

Background: Each year there are more than 800,000 deaths by suicide across the world, while India alone accounts for one third of female suicides and one fourth of male suicides worldwide. Responsible media reporting of suicide is an important suicide prevention intervention at the population level. There is sufficient evidence to show that the way suicide is reported and portrayed in the media can have a significant impact on individuals experiencing suicidal thoughts and behaviors. Recognizing the important role of the media in suicide prevention, the World Health Organization (WHO) issued guidelines for responsible reporting of suicides by the media. The Press Council of India, in 2019 endorsed WHO’s guidelines for media reporting of suicides, however there is no evidence that the Indian media is complying with these guidelines. Methods: To encourage responsible media reporting, we developed a scorecard to assess and rate media reports on suicide. We reviewed several resource documents that contained guidelines on responsible reporting of suicide. After consulting with a team of experts, we arrived at a scorecard that consisted of 10 positive and 10 negative parameters. Results: We applied the scorecard to 1318 reports on suicide from 9 English language newspapers, with the highest readership in India between the dates of 1 April to 30 June 2020. For the articles analyzed, the average positive score across all newspapers was 1.32 and the average negative score was 3.31. Discussion: The scorecard can be a useful tool to assess media reports on suicide and provide metrics for the same. It can facilitate improved monitoring and engagement with media organizations, who can quickly check their own reporting compliance to the WHO guidelines and compare how well they are performing compared to their peers over time.

## 1. Introduction

Worldwide, over 800,000 deaths by suicide take place annually [1]. In the year 2019, according to data from India’s National Crime Records Bureau [NCRB] there were 139,123 deaths by suicide, a 3.4% increase compared to 2018 [2]. While NCRB estimates India’s suicide rate at 10.4 per 100,000 population [2], tWorld Health Organization (WHO) estimates it to be nearly 58.6% higher at 16.5 per 1,00,000 of population [3], while a study based on Global Burden of Disease data estimated suicide rates to be nearly 72.1% higher at 17.9 per 100,000 population. Further, the study found that India accounted for 36.6% and 24.3% of global suicide deaths amongst women and men, respectively [4]. 

Irresponsible reporting of suicides by the media has been shown to be one of the many risk factors for suicide among populations, particularly in the youth [5,6]. There is a significant correlation between poor and sensationalist reporting of suicides and its consequent impact on individuals experiencing suicidal behavior and ideation and triggering imitative or copycat suicides [5,7,8,9,10]. Simultaneously, there is evidence to show that the media can be a protective factor as well. Responsible reporting of suicides can positively influence help-seeking behavior in vulnerable populations, contribute to awareness and shape attitudes about suicide [10,11].

There has been no conclusive research on the impact of media reporting of suicides on suicidal behavior among the Indian population. However, studies on the quality of media reporting on suicide in India have shown a poor standard of reporting, with minimal use of recommended protective reporting approaches [12], and significant disparities between epidemiological data on suicides and media reporting. Suicides involving females, younger people aged under 30 and those who were students or farmers were among those groups over-reported relative to their occurrence in the broader population, indicating that media determines which suicides are considered news-worthy [7]. Most suicide stories are reported by crime beat reporters who collect information from the police to produce routine and simplified incident report-style coverage of suicide incidents, and that graphic and sensational suicide reports are used as “clickbait” to generate audience interest [13].

Over the years, suicide prevention experts, international organizations and civil society groups have advocated for responsible reporting of suicides by the media. Recognizing the role of the media in suicide prevention, the WHO in 2008 published the Media Guidelines for reporting on suicide [14] which was later updated in 2017 based on a systematic review of over 100 research studies on the impact of media reporting on suicides [15]. The WHO guidelines for responsible reporting suggest practices which promote help-seeking behavior, increase awareness of suicide prevention, and provide alternative coping strategies for vulnerable readers [15]. Further, other public health agencies, such as the Centre for Disease Control and Prevention, USA recommend guidelines for media reporting of suicide as a part of larger suicide prevention strategies [16]. Civil society groups, such as the Samaritans, have developed resources for media professionals to promote the adoption of the recommended guidelines, while also advocating for ethical practices to be followed by the media in their coverage of suicide [17]. More recently, the International Association for Suicide Prevention (IASP) highlighted the urgent need for the adoption of guidelines on responsible reporting of suicides during the COVID-19 pandemic and suggested additional tips to support and supplement existing guidelines during the pandemic [3].

The Press Council of India (PCI) in 2019 issued a notification endorsing the WHO guidelines for responsible reporting of suicide, and drew upon the provisions on the Mental Health Care Act 2017 [18], which stipulates that the media should not publish photographs or any other information about a person undergoing mental health treatment without their consent [19]. While these guidelines do exist, there is evidence that media reporting is not concordant with these guidelines [18], due to various reasons including lack of awareness about the guidelines, poor implementation, and a deficit of monitoring mechanisms, to a limited understanding of the issue of suicide and how the media can contribute to suicide prevention efforts [17,20,21]. To increase awareness amongst media professionals about the existence of the guidelines and to encourage greater adherence to guidelines on reporting of suicides by the media, we developed a scorecard based on international (WHO) and national (PCI) guidelines for responsible reporting [18].This paper discusses the methodology used to develop the scorecard, and our findings from the assessment and scoring of suicide reports from English language newspapers, with the highest readership in India, between 1 April to 30 June 2020. This particular timeframe was chosen in-line with the quarterly calendar, and also owing to the concerns of increased incidences of suicides due to the onset of the COVID-19 pandemic. The purpose of such a scorecard is two-fold: to create an accessible tool that continuously assesses the performance of media publications in complying with guidelines on suicide reporting that could in addition be used as a functional checklist by journalists, and media professionals to ascertain whether their report on suicide meets a pre-defined set of guidelines. 

## 2. Methodology

The researchers were interested in creating an assessment tool to rate media reports using a numerical score that could be represented on a scale to assess adherence to media reporting guidelines on suicide and commonly used positive and negative practices. The scores for which would be simple to derive and straightforward to reproduce in intervals without highly trained human resources. In this way, the scores would be easy to communicate to journalists and media organizations and the tracking of their scores would highlight the progress made (or not) by these organizations in their adherence to media reporting guidelines. 

### 2.1. Review of Existing Assessment Tools on Suicide Reporting

The initial step in developing the scorecard was to determine if an evaluation tool exists that assesses media reporting of specific incidents of suicide against guidelines defined by suicide prevention experts. We conducted a narrative review of academic studies on suicide prevention and media reporting. Using PubMed, the researchers searched for the keywords “suicide,” “suicide prevention” and “media.” The query generated 636 results that were published between the years 2000 to 2020. After scanning through the abstracts of 37 relevant papers on media reporting and suicides (papers on evaluation and analyses (*n* = 10), guideline adherence studies (*n* = 10), reviews (*n* = 10), articles/editorials (*n* = 4), policy paper (*n* = 1)), we found only two assessment tools that assess the performance of newspapers in meeting guidelines on safe suicide reporting.

The first instrument developed by Nutt et al. is called the “Risk of Imitative Suicide Scale (RISc)”. While the scale is comprehensive in evaluating each article, it was developed to carry out a content analysis of each report to determine the density and saturation of negative and positive variables for each article. We felt it was impractical to apply such an instrument based on a substantial number of variables to large volumes of data on a repeated basis, to derive a score for a publication. We also felt the complexity of the scale in terms of calculating scores may make it inaccessible to media professionals and other concerned stakeholders who may be unfamiliar with the use of such scales [22].

The second instrument designed by John et al., called PRINTQUAL, examines the quality of newspaper reporting on suicides based on the guidelines published by Samaritans UK. We noticed there were a disproportionate number of parameters on the sub-scales; 19 on the negative scale on 4 on the positive scale. PRINTQUAL did use a simple scoring system that involved using a simple binary coding approach for each parameter on the article [23].

### 2.2. Development of Assessment Tool for Scoring Suicide Reports

The scorecard we set out to create drew from both instruments, however, would be far more accessible in terms of usage and comprehension. To develop such a tool, we chose to review guidance documents issued by the various national and international organizations including the WHO’s ‘Preventing suicide: a resource for media professionals’ guidelines (2017), the Centre for Disease Control and Prevention resource book titled ‘Preventing Suicide: A Technical Package of Policy, Programs, and Practices’ (2017), Samaritans’ ‘Media Guidelines for Reporting Suicide’ and the IASP briefing statement to highlight the urgent need for the adoption of guidelines on responsible reporting of suicides during the COVID-19 pandemic (2020). 

Most documents categorized guidelines as ‘Do’s and Don’ts’ for suicide reporting. The ‘Do’s’ comprised of protective factors that could be included by journalists in the content of the report. They mainly promote help-seeking behavior, educating readers on misconceptions about suicide and suicide prevention measures, demonstrate examples of overcoming suicidal thoughts and feelings, and ensure information provided is accurate and substantiated by an authority. The ‘Don’ts’ included practices that are likely to have an adverse effect on readers and were in clear violation of ethical norms related to suicide reporting and should therefore be avoided. The criteria could be associated with language, inappropriate content, images and positioning of the articles. For instance, language or phrases that sensationalize, criminalize or glamourize suicide, unnecessary focus on details such as the method, location or other information that fuels speculation about the suicide or attempted suicide, inconsiderate publishing of suicide notes or text messages related to the suicide or the inclusion provocative images of the scene of the suicide. In addition, as an extension of a study undertaken by authors G.A. and L.V., we also adapted several components of the coding frame that was previously applied on newspaper reports on suicide in Tamil Nadu and compiled by them among the other guidelines that were gathered during this review process [11].

We thus collated an extensive list of all commonly recommended guidelines from which 40 guidelines were identified based on the extent to which they impact individuals; both the subjects of the report and readers. The subjects of the report could be persons who may have attempted suicide or family, friends and relatives of the person who died by suicide or attempted suicide. Readers include individuals who are vulnerable or at risk of suicide at the time of reading the story as well as the general public who maybe unaware of the complexity of suicide and suicide prevention measures.

The “Do’s and Don’ts” helped us classify the guidelines into 19 positive and 21 negative criteria. The two lead researchers M.S. and T.F. further refined the scorecard by combining guidelines that converged or overlapped. For example, all criteria on confirming facts and evidence-based reporting were subsumed under a single criterion that is ‘facts verified by an official source.’ Further, we chose to exclude criteria whose interpretation could be influenced by the subjectivity of the investigator, such as use of sensationalizing language. 

We then applied these 40 guidelines to an initial dataset of specific reports on suicide. We gathered 20 reports from English-language newspapers with a minimum monthly readership of 1,500,000 [24]. Each report was coded based on the presence (coded a ‘1’) and absence (coded a ‘0’) of each guideline to determine the applicability of the identified guidelines to each article. Each guideline was summarized to ascertain how frequently it was observed in the data.

Thereafter, we conducted a consultation session with three suicide prevention experts, namely L.V., G.A. and S.P. to decide on criteria to include in the final scorecards. The shortlist derived was based upon (i) the significance of the criterion in impacting the reader: this included the potential positive impact of the criterion in promoting help-seeking behavior and reducing stigma associated with mental illness and suicides, as well as negative effects of the use of criminalizing language and publication of details that may lead to imitative suicides and violate the privacy of the individual and their bereaved family and friends; and, (ii) the frequency at which the criterion appeared in the preliminary dataset.

## 3. Results 

From an initial list of 40 criteria, we finally arrived at 20 top criteria for the scorecards, with 10 positive and 10 negative scoring criteria each (Table 1 and Table 2). 

Each criterion is scored as 1 (present) or 0 (absent) and all criteria have equal weightage to calculate the total positive and negative score for a media report on suicide. To arrive at a positive and negative score for each newspaper, the average score was calculated across all the reports for a given newspaper on both the positive and negative scale. On the positive scale, a score of 10 is the best score a newspaper can achieve while 0 is the worst. Inversely on the negative scorecard, a 10 is the worst and 0 is the best score of compliance with the reporting guidelines.

### Assessment of Reports on The Positive and Negative Scorecard

We selected nine of the highest read English language newspapers with a minimum monthly readership of 1,500,000 [24]. In addition to their wide reach, these newspapers were chosen as they were easily accessible online and were also in a language that the investigators involved in data collection and coding were comfortable with.

We included articles on specific cases of deaths by suicide or attempted suicide. The articles had to be in English and published between 1 April to 30 June 2020. We excluded articles that were general commentary pieces on suicide, suicide prevention and mental health; articles that solely focused on suicidal ideations; articles that were on bombings with an intent to kill oneself and others; reports where the cause of death was undetermined and could be a suicide, accidental death or homicide under investigation; and reports where less than 50% of the content of the article was on the death by suicide or suicide attempt. In such articles, there was insufficient data that could not be coded.

A team of trained researchers were involved in the data collection and analysis process. They identified articles from the newspapers assigned to them and collected case data and demographic data on the Media Reporting Scorecard. For gathering articles from newspapers, e-newspapers were combed through. In instances where the newspaper website offered a search function, articles were identified using the key words ‘suicide’, ‘kills self’, and ‘ends life’.

Subsequently, each article was independently coded on the positive and negative scale by following the Media Reporting Scorecard (see Table 1 and Table 2). The lead researchers, namely M.S. and T.F., convened periodically to discuss and resolve discrepancies in codes on the scoring scales. All discrepancies were addressed in this process and the researchers arrived at the final codes through concurrence. All the data was collated on to a master sheet and tabulated to provide positive and negative scores sorted by Newspaper and the proportion of reports that meet positive and negative parameters.

**Figure 1:**
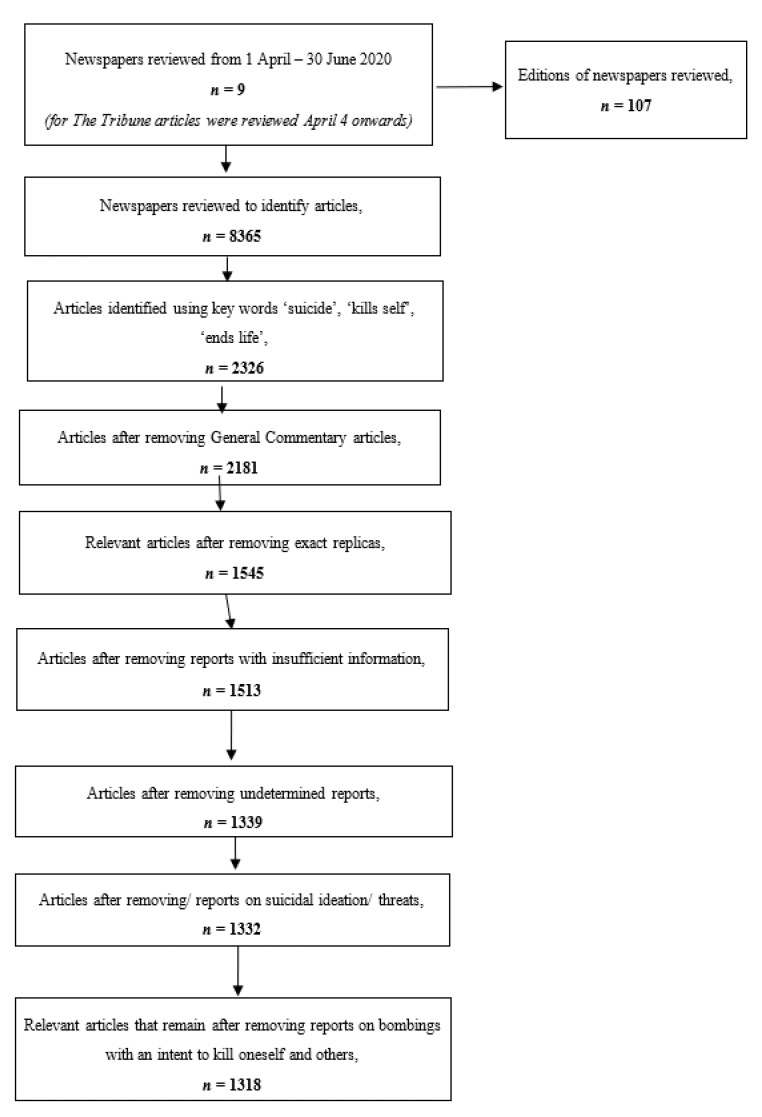
PRIMSA chart.

We applied the scorecard developed to articles on suicides and attempted suicides identified from nine newspapers and their 107 editions, between the dates of 1 April to 30 June 2020. We searched through 8365 daily newspapers, from which 2326 articles were identified, which were further screened to arrive at 1318 relevant articles on suicide and attempted suicides, which met the inclusion criteria. Details on the process to arrive at the 1318 articles can be found in Figure 1.

For the articles analyzed, the average positive score across all newspapers was 1.32 and the average negative score was 3.31. The average scores for newspapers on the positive and negative scorecard can be seen below in Table 3.

Details regarding the frequency at which each parameter was found to be present in the reports analyzed can be found in Table 4. The most common practices involved inclusion of verified information and facts from official sources (82.25% articles), followed by drawing linkages to poor mental health (21.7% articles). The practice of including verified facts and information is important since it is critical that facts of the case be correctly interpreted and reported.

Among negative reporting practices, the mention of the method of the suicide or attempted suicide (86.49% articles) and use of attention seeking headlines (75% articles) were the most common.

## 4. Discussion

The findings from the scorecards reveal that most newspapers performed poorly on both scorecards. On the negative scorecard, the average score of the newspapers was 3.31, which indicates a higher prevalence of negative reporting practices in the articles compared to positive practices which can be observed in the overall average positive score of 1.32. Only one criterion (verified information and facts from official source) was met by over 50% of articles on the positive scorecard. On the other hand, three criteria on the negative scorecard were coded in over 50% of articles: method of suicide mentioned in the articles (86.49%), attention-grabbing headlines (75.27%), and use of criminalizing language (65.17%). 

The results from the application of such a tool ha demonstrated the importance of a mechanism for monitoring adherence to media guidelines for reporting suicides. The scorecard has been designed and developed in a manner such that it can be adapted for different contexts. It could be translated to other languages as well as used across other platforms such as digital publications. The scorecard can be used as a means of monitoring the quality of articles on suicide and attempted suicides reported by the media or for monitoring specific criteria, such as the number of news reports providing help-seeking information. The disaggregated criteria data by newspaper helps in bringing to the attention of journalists and media professionals’ ways of improving their overall performance, functioning as a tool for self-regulation while reporting on suicides and attempted suicides. 

The strength of the scorecard developed is that it is easy use, can be adapted to different media formats and languages, and additionally can be used as an evidence-based resource for training media professionals on how to report responsibly on suicide, such that it complements larger suicide prevention efforts. Using the scorecard, each or all parameters can be monitored to track changes and trends in media reporting of suicides over time, particularly after events such as the death of a celebrity by suicide. For instance, whether the story of a celebrity death is repeated or published on the first page. The authors aim to disseminate the scorecard tool to media houses, representative associations and journalism training institutes to educate journalists, editors, students of journalism and other relevant stakeholders on the importance of the tool and its practical applications.

A limitation of the scorecard developed is that in order to ensure interrater reliability, each article needs to be independently coded twice by two researchers, who must then convene to address any discrepancies in the codes. To aid anyone using the scorecards to rate newspaper articles and avoid ambiguities while coding articles on the scorecard items, we also developed a guide with detailed descriptions of each item and identified key words for each of the parameters. The description of the parameters and some of the keywords identified can been seeing in Appendix A (Table A1 and Table A2). Further, the scorecard does not capture certain subjective parameters such as use of sensationalizing or glamourizing language as it introduces an element of researcher bias in interpreting the tone or language of an article. Another limitation of this study is that a narrative review was undertaken instead of a systemic review to evaluate existing tools for assessing media reporting of suicides, because of which there may have been some bias in identifying the studies for narrative review.

Despite these limitations, the scorecard can be used as an effective mechanism to monitor media reporting of suicides, and to encourage media publications to adopt guidelines for responsible reporting, in order to complement suicide prevention efforts and strategies.

Further, while the scorecard can be translated to other languages, challenges exist with regards to translating the scorecard and contextualizing it to the language of the region which will have to be explored. This will require working with experts in suicide reporting from the respective regions. We are currently working on methods to evaluate Indian language newspapers.

Going forward we intend to rate news reports on suicides each quarter, and use the data gathered to track any improvements or changes in trends of media reporting on suicide. The intention is to cover a larger number of digital publications and expand the application of the scorecard to assess reporting of suicides in non-English language publications. Newspapers and digital publication are only two mediums of news; there also exists several others like television, radio and social media, but presently there are no standardized guidelines to monitor the quality of reports on suicides and attempted suicides on these mediums. Assessing media reporting of suicides on other mediums of news would require tracking in real time which would require human and monetary resources.

The challenges posed by social media are particularly complex, since the platform enables the spread of information beyond news reports through comments sections, live streams and other public and private forums. Given the reach and impact of social media, a monitoring mechanism for reporting on suicides is imperative. Building on existing resources, new approaches should be developed and explored to regulate content on social media platforms which are constantly evolving and have a much wider reach compared to traditional sources of news such as newspapers and radio. Based on guideline issues, the parameters covered in the scorecard could be adapted to develop a similar mechanism for assessing adherence to positive practices in reporting of suicides.

## 5. Conclusions

Suicide prevention requires intersectoral collaborations and interventions. Responsible reporting of suicide by the media is a key universal suicide prevention strategy, which is yet to be fully adopted in countries, like India, where sensational reporting on suicides is rampant. Successful adoption of the media reporting scorecard developed by the authors can be used as a mechanism for monitoring media reporting of suicide, as well as be used as a tool for self-regulation by media professionals. Thereby reducing suicide rates among the general population, raising awareness among the public and encourage individuals to seek help.

## Figures and Tables

**Table 1:** Positive Scorecard.

No.	Positive Parameter	Description
1	Presence of help-seeking information	Provides information of national- or state-level support services that includes suicide prevention centers, emergency units in hospitals, 24/7 crisis helplines, self-help groups, mental health professionals, general physicians, community resources, rehabilitation centers.
2	Help-seeking information is up to date and operational	The information and contact details provided should be accurate and reliable. By operational we mean it should be relevant to where the article is published. This can be verified through a Google search.
3	Links to poor mental health	The report establishes a link between suicidal behavior and a mental illness, by making a clear reference to the individual’s struggle with a mental illness, its effect on their mental state. Inappropriate language such as “crazy” or “mental” is not accepted. Neither are mood qualifiers like “stressed”, “unhappy” etc. Acceptable terms that qualify include “depressed”, “anxious”, “panic”, “trauma”, “disturbed”, “distraught” or names of specific mental disorders.
4	Links to drug/ alcohol abuse	The report acknowledges the link between the suicide and substance and alcohol use. The report clearly refers to the person’s ongoing struggle with alcohol or drug addiction. A reference to the person’s intoxicated state at the time of suicide does not qualify.
5	Comments from mental health and suicide prevention experts	The article contains a quote or comment from a mental health professional or suicide prevention expert.
6	Reduces stigma highlights suicides are preventable	The article highlights that suicides are preventable by taking preventive measures and identifying risks in time and contains information that reduces stigma around talking about suicide and mental health concerns.
7	Credible population-level suicide statistics and/ or other research findings	The article reports on suicide-related statistical data. It may also provide findings from studies conducted on suicide and suicide prevention.
8	Challenges popular myths	The report challenges popular myths and reinforces their false nature. Examples of myths are (1) talking about suicide will lead to and encourage suicide; (2) people who talk about suicide do not mean to do it; (3) there are no preceding warning signs; and (4) there is nothing you can do to prevent suicide.
9	Links to hopeful stories	The report should have links to or snippets of reports that contain hopeful stories of people who have overcome suicidal thoughts and feelings.
10	Verified information and facts from official source	Information and facts are verified by official sources that include police officers, healthcare professionals or a government authority in the area. It has to be from a specific source if it simply says “sources”, that does not qualify. It could be likely that the information in the report is from an official source however, it may have not been acknowledged. It is important that reported information is corroborated, the absence of which may fuel speculation surrounding the story which in turn increases the likelihood of sensationalizing the report.

**Table 2:** Negative Scorecard.

No.	Negative Parameter	Description
1	Use of criminalizing language	The article uses phrases that associate suicide with a crime or sin, e.g., ‘committed suicide’.
2	Attention-grabbing headlines	The headline includes the word ‘suicide’, the method or the reason for the suicide
3	Method of suicide or attempted suicide is mentioned in the article	The article mentions the method of suicide or attempted suicide.
4	Describes method in detail	The article provides at least two specific details about the suicide/ attempted suicide method. The detail may include objects used in the suicide or specific names of substance used e.g., kerosene, ‘celphos’ tablets.
5	Discloses details of the suicide site	The report provides enough information to clearly identify the location and it is somewhat accessible to at least some members of the public.
6	Reduces reason to a single factor or event	The article clearly articulates that the suicide incident had just one motive, cause or trigger. It over-simplifies the complex realities of suicide by reducing it to a single factor. The causal relationship is NOT subject to speculation. Speculation about the cause is indicated by phrases such as ‘the reason may be’, ‘some uncertainties about the cause remain’.
7	Accompanying photos	The report publishes photographs or video footage of the deceased, bereaved members, the location of the suicide and the method as well as other dramatic/ emotional images (e.g., a noose, slit wrists, person standing on the ledge, etc.).
8	Contains information on grieving persons	The article reports on the effects of a suicide on bereaved persons or contains interview with bereaved persons. Relatives, friends of the victim and other private persons involved in the suicidal act or affected by the suicide are defined as bereaved persons.
9	Publishes note or text	The report publishes suicide notes, text messages, social media posts and emails of the deceased person and/or their family members.
10	Article is on the front page of the newspaper	The article is published on the first page. This includes articles that commence on the first page and are then continued in later pages.

**Table 3:** Average positive and negative scorecard.

Newspaper Name	Positive Score	Negative Score
The Times of India	1.12 (0.66) *	3.37 (1.27)
Hindustan Times	1.31 (0.69)	3.28 (1.22)
The New Indian Express	1.07 (0.91)	3.42 (1.18)
The Telegraph	1.03 (0.55)	3.37 (1.31)
The Hindu	2.71 (0.92)	2.35 (1.15)
The Indian Express	1.32 (0.68)	3.32 (1.33)
Mirror	1.32 (0.73)	4.13 (1.35)
The Tribune	1.00 (0.62)	4.04 (0.98)
The Economic Times	1.13 (0.83)	3.25 (1.83)
Total	1.32 (0.87)	3.31 (1.31)

* Values are presented as Mean (SD).

**Table 4:** Number and proportion of total articles that meet positive and negative scoring criteria.

Positive Scoring Criteria		No. of Articles
1	Verified information and facts from official source	1084 (82.25%)
2	Links to poor mental health	286 (21.70%)
3	Presence of help seeking information	139 (10.55%)
4	Help-seeking information is up to date and operational	139 (10.55%)
5	Links to drug/alcohol abuse	61 (4.63%)
6	Comments from mental health and suicide prevention experts	13 (0.99%)
7	Credible population-level suicide statistics	6 (0.46%)
8	Reduces stigma highlights suicides are preventable	4 (0.30%)
9	Challenges popular myths	2 (0.15%)
10	Links to hopeful stories	0 (0.00%)
	**Negative Scoring Criteria**	**No. of Articles**
1	Method of suicide or attempted suicide is mentioned in the article	1140 (86.49%)
2	Attention-grabbing headlines	992 (75.27%)
3	Use of criminalizing language	859 (65.17%)
4	Reduces reason to a single factor or event	541 (41.05%)
5	Discloses details of the suicide site	228 (17.05%)
6	Describes method in detail	194 (14.72%)
7	Accompanying photos	148 (11.23%)
8	Contains information on grieving persons	126 (9.56%)
9	Article is on the front page of the newspaper	79 (5.99%)
10	Publishes note, text and social media post	61 (4.63%)

## Data Availability

The datasets will be made available to appropriate academic parties on request from the principal investigator in accordance with the data sharing policies of the institute.

## Appendix A

**Table A1:** Description of parameters and keywords identified for the Positive Scorecard.

Positive Scorecard		
Positive Parameter	Description	Example
Presence of help seeking information	Provides information of national or state-level support services that includes suicide prevention centers, emergency units in hospitals, 24/7 crisis helplines, self-help groups, mental health professionals’ general physicians, community resources, rehabilitation centers.	“Those in distress or having suicidal tendencies could seek help and counselling by call Sneha - 044- 24640050”
Information is up to date and operational	The information and contact details provided should be accurate and reliable. By operational we mean it should be relevant to where the article is published. This can be verified through a Google search.	Placed at the bottom of the article.
Links to poor mental health	The report establishes a link between suicidal behavior and a mental illness, by making a clear reference to the individual’s struggle with a mental illness, its effect on their mental state. Inappropriate language such as “crazy” or “mental” is not accepted. Neither are mood qualifiers like “stressed”, “unhappy” etc.	“Before ending his life, the deceased, who was said to be depressed”
Links to drug/ alcohol abuse	The report acknowledges the link between the suicide and the substance and alcohol use. The report clearly refers to the person’s ongoing struggle with alcohol or drug addiction. A reference to the person’s intoxicated state at the time of suicide does not qualify.	“Owing to the liquor ban, Suneesh was in a state of alcohol withdrawal and took his life”
Comments from mental health and suicide prevention experts	The article contains a quote or comment from a mental health professional or suicide prevention expert.	“Dr. Singh, a psychiatrist at KEM Hospital encourages reaching out to family members or friends who are disconnected, alone and have expressed distress.”
Reduces stigma highlights suicides are preventable	The article highlights that suicides are preventable by taking preventive measures and identifying risks in time and contains information that reduces stigma around talking about suicide and mental health concerns.	“Suicides can be prevented and it’s okay to talk about how you are feeling,”
Credible population-level suicide statistics and/ or other research findings	The article reports on suicide-related statistical data. It may also provide findings from studies conducted on suicide and suicide prevention.	“The Ministry of Agriculture reported 127 farmers who died by suicide during the period of the lockdown.”
Challenges popular myths	The report challenges popular myths and reinforces false nature of such myths: Talking about suicide will lead to and encourage suicide. People who talk about suicide do not mean to do it. People with mental illnesses usually are the ones who end their lives. There are no preceding warning signs. There is nothing you can do to prevent suicide.	“Socially, mental illness and thoughts about suicide are not something we talk about… what we have learnt from the mental health partners and academics we have worked with is that being connected is a protective factor in suicide prevention.”
Links to hopeful stories	The report should have links to or snippets of reports that contain hopeful stories of people who have overcome suicidal thoughts and feelings.	“Mohammad Shami has spoken about suicidal thoughts he faced in the past and has opened up about the help he received to overcome those thoughts in this article.”
Verified information and facts from official source	Information and facts are verified by official sources that include police officers, healthcare professionals or a government authority in the area.	“Police confirmed, Harish jumped off the bridge at 1am last night”

**Table A2:** Description of parameters and keywords identified for the Negative Scorecard.

Negative Scorecard		
Negative Parameter	Description	Example
Use of criminalizing language	The article uses phrases that associates suicide with a crime or sin, ‘committed suicide’.	“He committed suicide by jumping off the balcony from his residence”.
Attention-grabbing headlines	The headline includes the word ‘suicide’, mentions a life event, the method or the reason for the suicide or attempted suicide.	“Man jumps to death on testing positive for COVID-19” “Woman commits suicide by hanging after marriage failure”
Describes method in detail	The article provides at least two specific details about the suicide/ attempted suicide method.	“… jumped from the third floor balcony by hoisting themselves off the grill” “… attempted to hang herself from a ceiling fan using her dupatta”
Method of suicide or attempted suicide is mentioned in the article	The article mentions the method of suicide or attempted suicide.	“He was found hanging in his room.”
Discloses details of the suicide site	The report provides enough information to clearly identify the location and it is somewhat accessible to at least some members of the public.	“The deceased took their life in their residence at Prestige Luxe Complex in Koramangala.” “After her test results came back negative, she took her life by jumping from the window of KEM Hospital.”
Reduces reason to a single factor or event	The article clearly articulates that the suicide incident had just one motive, cause or trigger. It over-simplifies the complex realities of suicide by reducing it to a single factor.The causal relationship is NOT subject to speculation.	“Upon losing his job, Prakash ended his life.” “Police said that he was under severe financial stress and therefore took this extreme step.”
Accompanying photos	The report publishes photographs or video footage of the deceased, bereaved members, the location of the suicide and the method as well as other dramatic/emotional images (e.g., a noose, slit wrists, person standing on the ledge, etc.).	
Contains information on grieving persons	The article reports on the effects of a suicide on bereaved persons or contains interview with bereaved persons. Relatives, friends of the victim and other private persons involved in the suicidal act or affected by the suicide are defined as bereaved persons.	“After no response from banging her door, her father kicked down the door and was shocked to find her hanging from the fan.”
Publishes note, text and social media post	The report publishes suicide notes, text messages, social media posts and emails of the deceased person and/or their family members.	“In his suicide note, the deceased said “I am ending my life because I see no point in living. No one is to blame for my death.”
Article is on the front page of the newspaper	The article is published on the first page. This includes articles that commence on the first page and are then continued in later pages.	
